# Dietary spirulina supplementation modifies rumen development, fermentation and bacteria composition in Hu sheep when consuming high-fat dietary

**DOI:** 10.3389/fvets.2023.1001621

**Published:** 2023-01-30

**Authors:** Zhibo Wang, Yaxu Liang, Jiawei Lu, Zongyou Wei, Yongjin Bao, Xiaolei Yao, Yixuan Fan, Feng Wang, Daxiang Wang, Yanli Zhang

**Affiliations:** ^1^Institute of Goats and Sheep Science, Nanjing Agricultural University, Nanjing, Jiangsu, China; ^2^Agricultural and Rural Science & Technology Service Center, and Enterprise Graduate Workstation, Taicang, China; ^3^Jiangsu Qianbao Animal Husbandry Co., Ltd, Yancheng, Jiangsu, China

**Keywords:** rumen microbiota, Hu sheep, rumen morphology, spirulina supplementation, rumen development and fermentation

## Abstract

**Introduction:**

This study aims to investigate the long-term effects of spirulina supplementation in a high-fat diet (HFD) on rumen morphology, rumen fermentation, and the composition of rumen microbiota in lambs. Spirulina is a blue-green microalgae that has been shown to have high nutritional value for livestock.

**Methods:**

Fifty-four lambs were randomly divided into three groups: a normal chow diet (NCD) group, a high-fat diet (HFD) group, and a high-fat diet supplemented with 3% spirulina (HFD+S) group. Rumen morphology, rumen fermentation, and rumen microbiota were analyzed at the end of the study.

**Results:**

Spirulina supplementation improved the concentration of volatile fatty acids and rumen papilla length. Additionally, there was a tendency for an increase in rumen weight and an upregulation of the genes *Claudin-1, Claudin-4*, and Occludin in the HFD+S group. Pyrosequencing of the 16S ribosomal RNA gene also showed that spirulina supplementation significantly changed the rumen microbiota composition in the HFD group, with a decrease in richness and diversity. Specifically, the relative abundance of *Prevotella 9* and *Megasphaera* was significantly increased in the HFD group compared to the NCD group, while spirulina supplementation reversed these changes.

**Discussion:**

This study suggests that 3% spirulina supplementation can improve rumen development and fermentation, and effectively relieve rumen microbe disorders in lambs caused by a high-fat diet. However, further research is needed to confirm the findings and to examine the long-term effects of spirulina supplementation in different types of livestock and under different dietary conditions.

## Introduction

Spirulina is a functional additive that contains several active components, such as phenolic acids, beta-carotene, vitamins, minerals, tocopherols, fatty acids, and gamma-linolenic acid (1, 2). It belongs to the Oscillatoriaceae family. It is also rich in antioxidants, including essential amino acids (3–5). Spirulina also reduce the blood lipid content through its rich content of gamma-linolenic acid (6). Based on these nutritional benefits, spirulina is now used as a food supplement for both humans and livestock. Spirulina can be used as an amino acid supplement in poultry and piglet diets and is effective in relieving sows from nutritional metabolism disorders due to gestation (7–9). A previous study showed that it was beneficial to lamb growth when they were fed 20% spirulina supplementation (10). In addition, our previous study found that adding 3% spirulina to a high-energy diet could improve the immune and antioxidant capacity of sheep and alleviate lipid metabolism disorder (6). Researchers have also considered using spirulina to reduce obesity-associated chronic inflammatory states (11–13). However, the effects of spirulina in the diet on rumen microbiota remain unknown.

The rumen development of lambs can be divided into three stages: the non-rumination stage from birth to 3 weeks of age, the transition stage from 3 to 8 weeks of age, and the rumination stage after 8 weeks of age (14). The morphological development of rumen epithelium in ruminants may be accompanied by molecular adaptations of nutrient absorption and metabolism (15). The diversity of the rumen microbial community is one of the main ways to understand rumen function (16). Microbes are important for animal productivity because they help degrade carbohydrates, which are then turned into volatile fatty acids (VFA) to supply energy for metabolic functions (17). The rumen microbiota also plays a vital role in the metabolism of fatty acids in dietary fat (18, 19).

According to the previous results of our team, high fat diet has significant effect on growth performance in Hu sheep. High fat diet obviously downregulated average daily feed intake and feed/gain ratio whereas upregulated the GR value (6). Some studies have connected a high-fat diet to the development of gastrointestinal diseases (20, 21). However, whether spirulina supplementation can ameliorate the negative effects of rumen morphology and the ruminal bacteria imbalance caused by consuming high-fat diets remains poorly understood. Therefore, the effects of dietary spirulina supplementation on rumen morphology, fermentation, and microbiota composition were examined in Hu sheep fed an HFD diet.

## Materials and methods

### The management of animals and experimental design

All animal experimental procedures were approved by the Ethics Committee of Nanjing Agricultural University, China (Approval ID: SYXK2011-0036). A total of 54 male lambs without castration (27.5 ± 1.78 kg) at 3 month of age were raised at Qidong Ruipeng Animal Husbandry, Jiangsu Province, China. The lambs were placed into three random groups: a normal chow diet (NCD), high-fat diet (HFD), and high-fat diet supplemented with 3% spirulina (HFD + S). There were three replicates per group and six lambs per replicate. Three percent fat was added to the high-fat diet to increase the energy level. The floors, walls, and fences of the lamb house were disinfected. Vaccination, parasites, and other prophylactic measures were carried out during the prefeeding to make sure that the lambs were healthy. The experiment continued for 74 days, including a 14 days adaptation period. The feed formulation described by Liang et al. (6) was carried out and is presented in Table 1. Powdered spirulina was purchased from a commercial supplier (Ordos Mengjian Spirulina Co., Ltd., Inner Mongolia, China).

**Table 1:** Diet ingredients and chemical composition from Hu sheep fed standard (ST) and high-energy (HE) diets divided in supplemented subgroups: control and spirulina experimental diets.

**Items**	**Spirulina**	**NCD**	**HFD**	**HFD + S**
**Diet ingredients, % DM**				
Corn		35	44	45
Fat powder		0	3	3
Malt root		3	1.5	1
Soybean meal		10	12	8
Corn germ meal		9	8.5	9
Rice husk		5	3	4
Soybean husk		5	3	2
*Pleurotus eryngii* residue		5	5	5
Corn husk		18	15	15
Rice bran		5	0	0
Spirulina		0	0	3
Premix^a^		5	5	5
Total		100	100	100
**Chemical composition**				
DE, MJ/kg	18.1	11.7	13.0	13.1
ME, MJ/kg	16.0	10.0	11.0	11.1
Dry matter, %	93.6	92.5	91.9	92.3
Organic matter, % DM	93.1	92.1	92.8	94.2
Crude protein, % DM	60.0	12.9	12.9	13.0
Ether extract, % DM	5.0	6.0	7.2	7.8
NDF, % DM	15.9	38.4	32.2	30.0
ADF, % DM	0.0	18.3	13.7	12.7

DM, dry matter; DE, digestible energy; ME, metabolic energy; NDF, neutral detergent fiber; ADF, acid detergent fiber. ^a^The premix provided the following per kg of diet: vitamin A, 3000 IU; vitamin D3, 600 IU; vitamin E, 6 mg; Cu, 11 mg; copper, 11.0 mg; Fe, 40.0 mg; Mn, 50.0 mg; Zn, 50.0 mg; Se, 0.15 mg; Co, 0.5 mg; I, 0.4 mg; N, 0.2 g; lysine, 0.025 g.

### Sample collection

After 2 months of feeding, five Hu sheep with similar body weights and body conditions were randomly selected from each group and slaughtered without feeding. Immediately after slaughter, a representative sample of rumen digestive fluid (at least 200 ml) was collected to determine the pH value of rumen fluid by pH meter (Code: fc230b, Hanna, Italy). Then the rumen fluid was filtered through four layers of cheesecloth and stored at −20°C for VFA concentration analysis (22). Rumen tissues were collected and fixed with 4% paraformaldehyde for histomorphological analysis. At the same time, the rumen digesta samples were thoroughly mixed well, collected into a 5 ml cryopreservation tube, and stored at −80°C for further analysis.

### Analysis of rumen tissue morphology and rumen fermentation parameters

The VFA concentration in the rumen fluid was analyzed according to the method reported by Feng and Gao (23). Gas chromatography (GC-14B; Shimadzu, Japan; capillary column film thickness: 30 m × 0.32 mm × 0.25 μm; column temperature = 130°C; injector temperature = 180°C; detector temperature = 180°C) was used to determine VFA, which has been used in prior experiments (24). Crotonic acid was used as the internal standard.

The morphology of the rumen was assessed using the method described by Ye et al. (25). Rumen tissues were washed in phosphate buffer saline (PBS) and fixed in 4% formaldehyde before being embedded in paraffin and stained with hematoxylin and eosin. With 40 × objective lens, measurements of lesions were taken. Each lamb sample in one group received five slides, with each slide capturing two photographs. We measured the predefined criteria described earlier, using Image Pro Plus software (Media Cybernetics, Bethesda, MD, USA) (26). During the analysis, three extreme samples were removed from each group, and there were altogether 36 samples.

### DNA extraction and high-throughput sequencing

The lamb rumen content sample was collected for microbial profile analysis and stored at −80°C until analysis. The DNA was isolated using MN NucleoSpin 96 Soi (MN-MACHEREY-NAGEL, Germany). With the primers 338F (5′ ACTCCTACGGGAGGCAGCA-3′); 806R (5′-GGACTACHVGGGTWTCTAAT-3′) and the cycling settings used by Hu et al. (22), the V3–V4 regions of the bacterial 16S RNA gene were amplified. PCR products were sequenced on the Illumina MiSeq platform by high-throughput pyrosequencing at Biomarker Technologies Co., Ltd. (Beijing, China). Trimmomatic V0.33 software filtered the raw reads obtained by sequencing. Cutadapt 1.9.1 software was used to identify and remove primer sequences, and clean reads without primer sequences were obtained. To acquire the final effective readings, they were grouped into operational taxonomic units with 97% similarity (OTU). The chimeric sequences were identified and deleted using the UCHIME V4.2 program.

### RNA isolation, cDNA synthesis and qPCR

The Trizol method described by Liang was used to extract total RNA from ruminal tissue (6). The RNA concentration was then quantified using a NanoDrop 2000 Spectrophotometer (Thermo Scientific, Waltham, MA, USA). The absorption ratio (260/280 nm) of all samples was between 1.8 and 2.1, indicating high RNA purity. Total RNA was used for reverse transcription using a PrimeScript^®^ RT reagent kit with gDNA Eraser (Takara Bio, Otsu, Japan). The expression of the target genes was determined using the QuantStudio 5 Real-time PCR Instrument (Applied Biosystems, Foster, California, USA) with fluorescence detection of AceQ qPCR SYBR Green Master Mix (Vazyme Biotech, Nanjing, China) under the standard program. The data of the gene expression were normalized by the housekeeping gene (actin beta, ACTB) using the 2^−Δ*ΔCT*^ method. The primers and amplicon sizes of the genes are shown in Supplementary Table S1.

### Statistical analysis

The basic data (rumen papillae, thickness, total volatile fatty acid content and pH) were subjected to one-way analyses of variance (ANOVA) run using Duncan's test. The data were processed as mean values ± SEM. *P* < 0.05 was considered to be significantly different. The analysis was performed using SPSS 25.0 (IBM Corp., Armonk, NY, USA).

## Results

### Effect of spirulina supplementation on rumen development of Hu sheep fed with HFD

**Figure 1:**
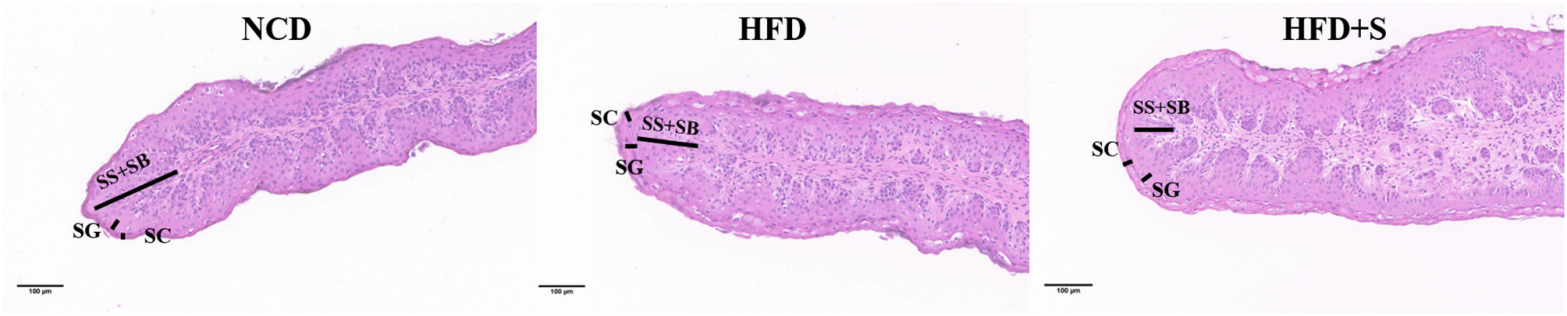
Hematoxylin and eosin staining (HE) sections of lambs' rumen papillae tissues.

The rumen development characteristics of Hu sheep fed different diets are shown in Figure 1 and Table 2. Compared with the HFD group, there was no significant difference in rumen weight (emptied rumen weight and relative weight) between the NCD group and the HFD + S group, but there was an increasing trend (*P* > 0.05). However, the ruminal papilla length (*P* < 0.05), the thickness of total epithelial (*P* < 0.05), and stratum corneum (SC, *P* < 0.05) in the HFD group were significantly lower than those in the other two groups. The thickness of stratum granulosum (SG, *P* < 0.001) in the NCD group were significantly higher than other two groups, but the thickness of stratum spinosum and basale were not significantly different among the three groups (SS + SB, *P* > 0.05; Table 2).

**Table 2:** Effect of spirulina supplementation on the growth and development of rumen and rumen papillae of Hu sheep fed HDF.

**Items**	**Groups**			***P*-value**
	**NCD**	**HFD**	**HFD** + **S**	
Emptied rumen weight, g	0.92 ± 0.12	0.70 ± 0.10	0.82 ± 0.13	0.094
Rumen relative weight (body weight%)	2.21 ± 0. 24	1.69 ± 0.27	1.83 ± 0.17	0.070
Rumen papillae length, mm	2,861.02 ± 668.44^a^	1,906.31 ± 358.18^b^	2,285.49 ± 179.16^a^	0.043
Total epithelia thickness, μm	142.87 ± 27.82^a^	117.08 ± 20.22^b^	124.42 ± 13.54^a^	0.019
Stratum corneum thickness, μm	18.48 ± 5.07^a^	10.83 ± 2.32^b^	15.02 ± 5.90^a^	0.002
Stratum granulosum thickness, μm	15.36 ± 3.29^a^	10.34 ± 1.88^b^	10.62 ± 2.13^b^	< 0.001
Stratum spinosum and basale thickness, μm	109.02 ± 23.91	94.26 ± 17.50	98.78 ± 15.97	0.190

In the same row, values with different small letter superscripts indicate significant difference (P < 0.05).

### Effect of spirulina supplementation on rumen fermentation of Hu sheep fed with HFD

The phenotypic characteristics of rumen fermentation in Hu sheep in the three groups are shown in Table 3. Compared whith the NCD group, HFD treatment significantly decreased overall VFA, acetate, propionate, and butyrate concentrations while significantly increasing the acetate to propionate ratio (*P* < 0.05). Furthermore, spirulina supplementation could ameliorate the effect of an HFD on rumen fermentation. There were no significant variations in the pH value or the concentrations of isobutyrate, valerate, and isovalerate among the three groups (*P* > 0.05).

**Table 3:** Effect of spirulina supplementation on rumen fermentation parameters of Hu sheep fed HFD.

**Items**	**Groups**			***P*-value**
	**NCD**	**HFD**	**HFD** + **S**	
pH	6.75 ± 0.32	7.07 ± 0.22	6.75 ± 0.49	0.318
Acetate, mmol/L	20.97 ± 0.87^a^	11.68 ± 3.45^b^	18.28 ± 3.90^a^	0.005
Propionate, mmol/L	14.35 ± 3.07^a^	3.90 ± 0.65^c^	9.42 ± 3.37^b^	0.001
Butyrate, mmol/L	3.90 ± 0.56^a^	1.38 ± 0.42^b^	3.49 ± 1.23 ^a^	0.019
Isobutyrate, mmol/L	0.31 ± 0.10	0.39 ± 0.21	0.47 ± 0.21	0.291
Valerate, mmol/L	0.72 ± 0.29	0.37 ± 0.01	0.63 ± 0.30	0.165
Isovalerate, mmol/L	0.46 ± 0.10	0.86 ± 0.56	0.97 ± 0.54	0.291
Total VFA, mmol/L	40.17 ± 3.34^a^	18.78 ± 4.41^b^	32.73 ± 8.96^a^	0.002
A/P	1.55 ± 0.44^b^	2.98 ± 0.65^a^	2.01 ± 0.29^b^	0.007

In the same row, values with different small letter superscripts indicate significant difference (P < 0.05).

### Effect of spirulina supplementation on rumen microbiota diversity of Hu sheep fed with HFD

**Figure 2:**
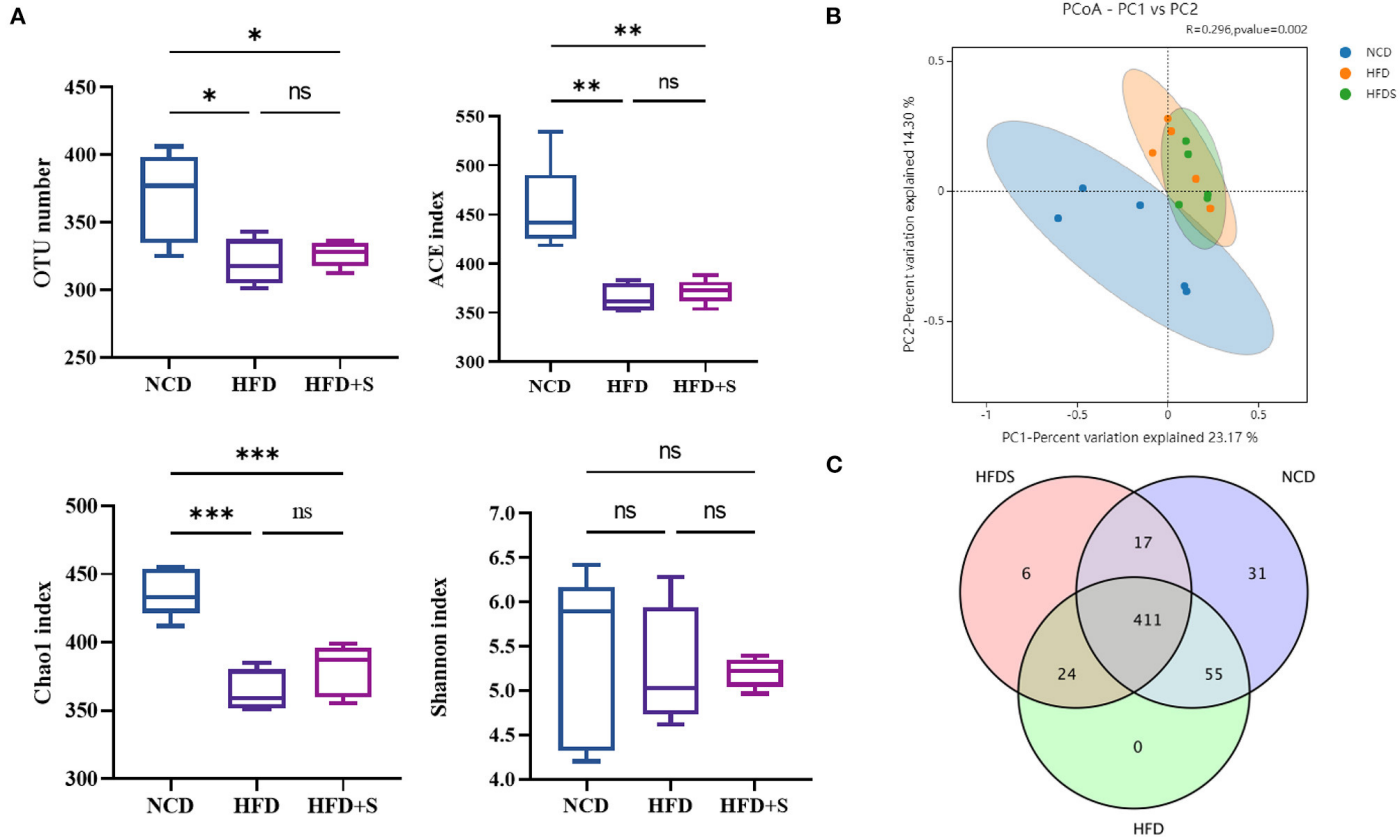
Effect of high-fat diet and spirulina supplementation on the rumen bacterial communities' diversity. **(A)** Comparison of the OUT number, ACE, Chao1 and Shannon of the α-diversity in NCD, HFD and HFD + S groups. **(B)** Bacterial communities PCoA based on the OUT level. **(C)** Based on the OUT level, Venn diagrams. “^*^” represents a significant correlation (*P* < 0.05), “^**^” represents an extremely significant correlation (*P* < 0.01), “^***^” represents an extremely significant correlation (*P* < 0.001).

To examine the effect of spirulina supplementation on ruminal microbiota, ruminal bacterial communities were determined by Illumina HiSeq sequencing of the 16S RNA V3–V4 region. As shown in Supplementary Table S2, 1,198,763 raw reads were obtained from the high-throughput sequencing library. Clean reads were obtained through quality filtering using QIIME1, 1,178,220. A total of the effective read clustered into operational taxonomic units (OTUs), and OTUs were formed at the 97% similarity level. All the rarefaction curves tended to approach the plateau (Supplementary Figure S1). Notably, the analysis of OTUs number (*P* = 0.0149), ACE (*P* = 0.0011) and Chao1 (*P* = 0.0001) indicated that rumen bacterial diversity was reduced in both HFD and HFD + S groups (Figure 2A). Furthermore, based on the analysis of Bray-Curits Metric and Principal coordinates (*R* = 0.296, *P* = 0.002), the difference in rumen microbiota composition was found among the three groups (Figure 2B). Furthermore, a Venn diagram analysis showed 411 common core OTUs in the three groups, as well as 31 and 6 unique OTUs in the NCD and HFD groups, respectively (Figure 2C). These findings showed that HFD feeding affected the microbiological composition of rumen contents, but HFD + S had no effect on their species (*P* > 0.05).

### Effect of spirulina supplementation on the bacterial composition of Hu sheep fed HFD

**Figure 3:**
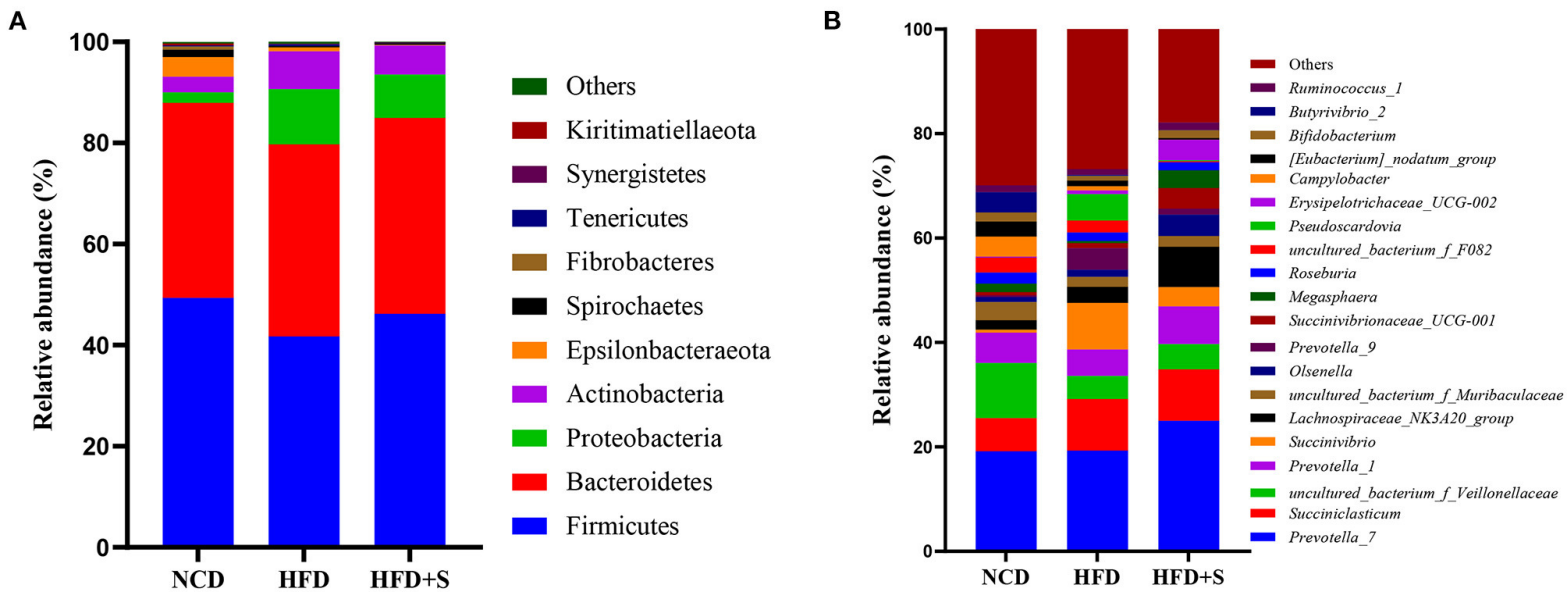
The taxonomic profiles for the relative abundance in the ruminal digesta of each group. **(A)** The relative phylum-level abundance in the ruminal digesta. **(B)** The relative genus-level abundance in the ruminal digesta.

A total of 18 phyla and 158 genera were found in the rumen microbiota. As shown in Figure 3A, at the phylum level, four major dominant phyla were identified in three groups: *Firmicutes, Bacteroidetes, Proteobacteria* and *Actinobacteria*. The phylum level analysis indicated that HFD and HFD+S feeding obviously increased the relative abundance of *Proteobacteria* and decreased *Firmicutes* level (*P* < 0.05), while spirulina supplementation significantly ameliorated the influences of these bacteria caused by HFD treatment (*P* < 0.05). To further identify the differences among each group, a genus-level analysis was performed. As observed in Figure 3B, the relative abundance of *Prevotella_9* and *Megasphaera* significantly increased in the HFD group compared with the NCD and HFD + S groups (*P* < 0.05).

### Effect of spirulina supplementation on rumen microbiota's key phylotypesbacteria composition in HFD-fed sheep

**Figure 4:**
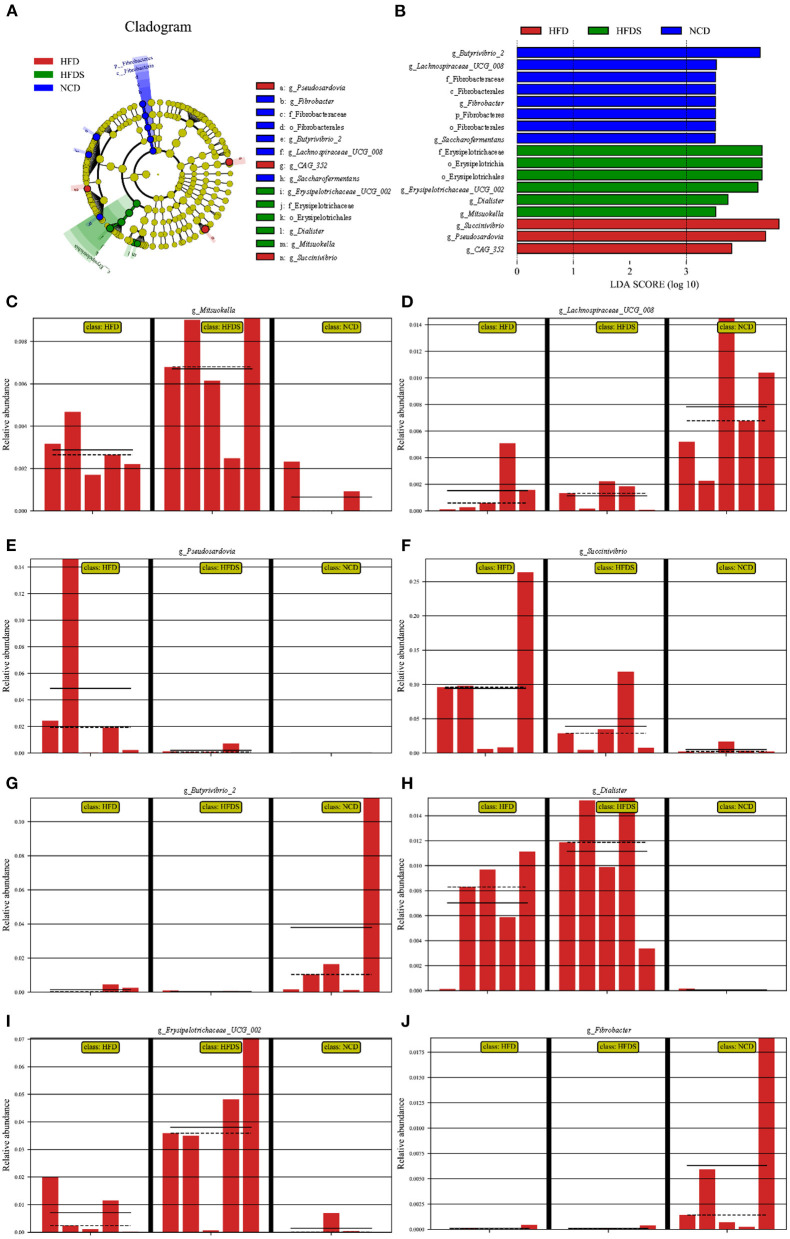
High-fat feeding and spirulina supplementation are associated with multiple bacteria. **(A)** The taxonomic cladogram was obtained from LEfSe analysis. **(B)** Linear discriminant (LDA) and effect size (LEfSe) analysis of the three groups. **(C–J)** The relative abundance of bacterial obtained in rumen microbiota from the LefSe results.

The OTUs of each group were compared to identify the significantly abundant bacterial groups under different diet treatments. Biomarker detection was performed using linear discriminant analysis (LDA) LEfSe with a 3.5-threshold value at the genus level (Figures 4A, B). The relative abundance of *Butyrivibrio_2, Lachnospiraceae_UCG_008, Fibrobacter*, and *Saccharofermentans* were significantly increased in the NCD group; *Succinivibrio, CAG_352*, and *Pseudoscardovia* were significantly increased in the HFD group; *Erysipelotrichaceae_UCG_002, Dialister*, and *Mitsuokella* were significantly increased in the HFD + S group (Figures 4C–J).

### The relationship between the ruminal bacterial community and fermentation parameters in Hu sheep

**Figure 5:**
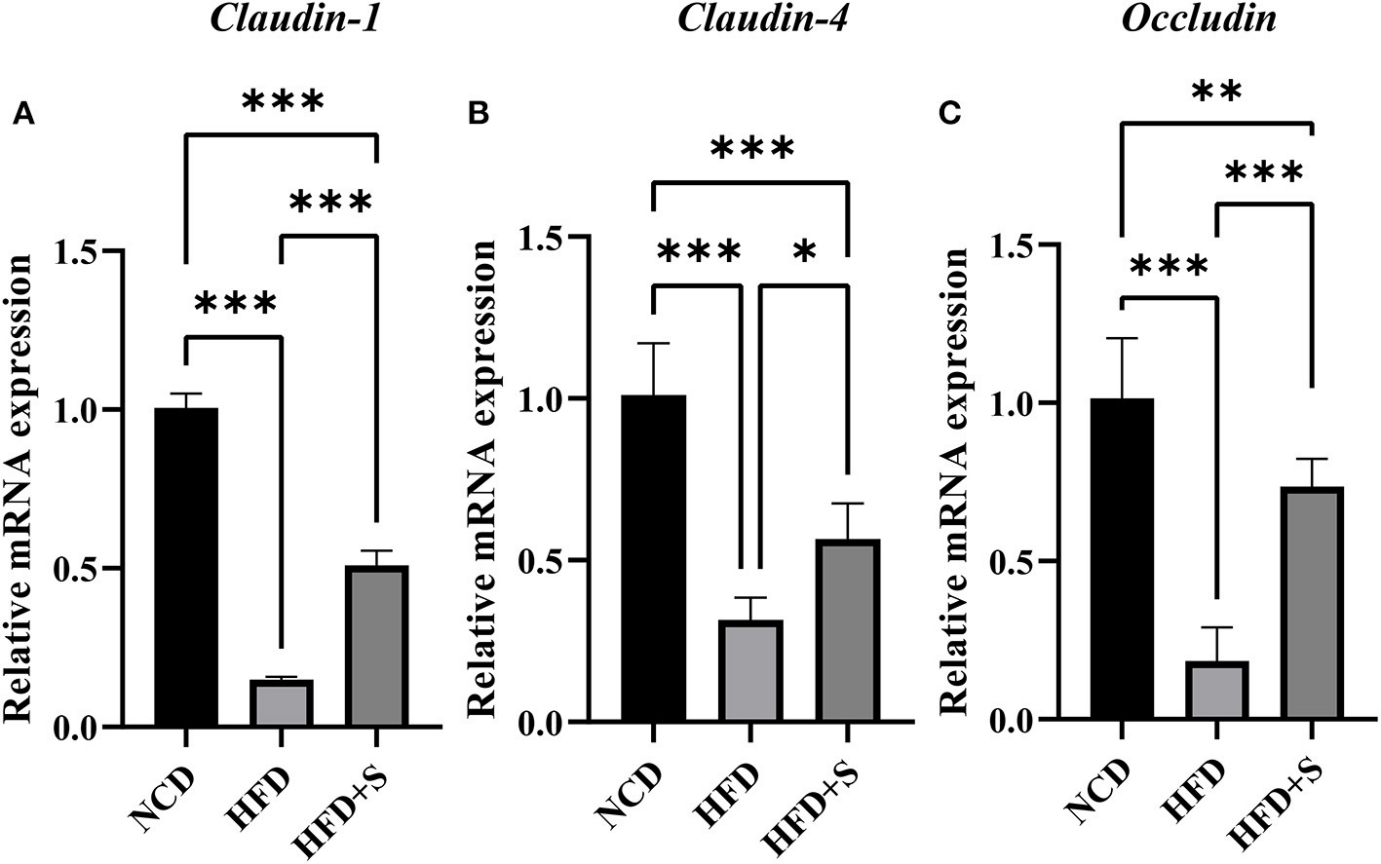
**(A–C)** Relationship between changes of microbial community and fermentation parameters of the ruminal digesta. “^*^” represents a significant correlation (*P* < 0.05), “^**^” represents an extremely significant correlation (*P* < 0.01), “^***^” represents an extremely significant correlation (*P* < 0.001).

The concentrations of acetate, propionate, isobutyrate, butyrate, isovalerate, valerate, total VFA, and the acetate to propionate ratio in the rumen liquid were measured to assess the relationship between the top 15 relative abundance ruminal bacterial communities and the composition of VFA in the rumen liquid. We found five significant positive correlations, four significant negative correlations, and three highly significant negative correlations in the correlation analysis between the ruminal bacterial community and the fermentation parameters. *Prevotella_1* had a significantly negative correlation with valerate content (*P* < 0.05); *Succinivbrio* had a negative correlation with total VFA, acetate, and propionate concentrations (*P* < 0.05); *Megasphaera* and *Erysipelotrichaceae*_*UCG-002* had a significantly positive correlation with isovalerate concentration (*P* < 0.05); *Ruminococcus_1* had a negative correlation with butyrate and valerate concentrations (Figure 5).

### Effect of spirulina supplementation on rumen tight junction proteins of Hu sheep fed HFD

**Figure 6:**
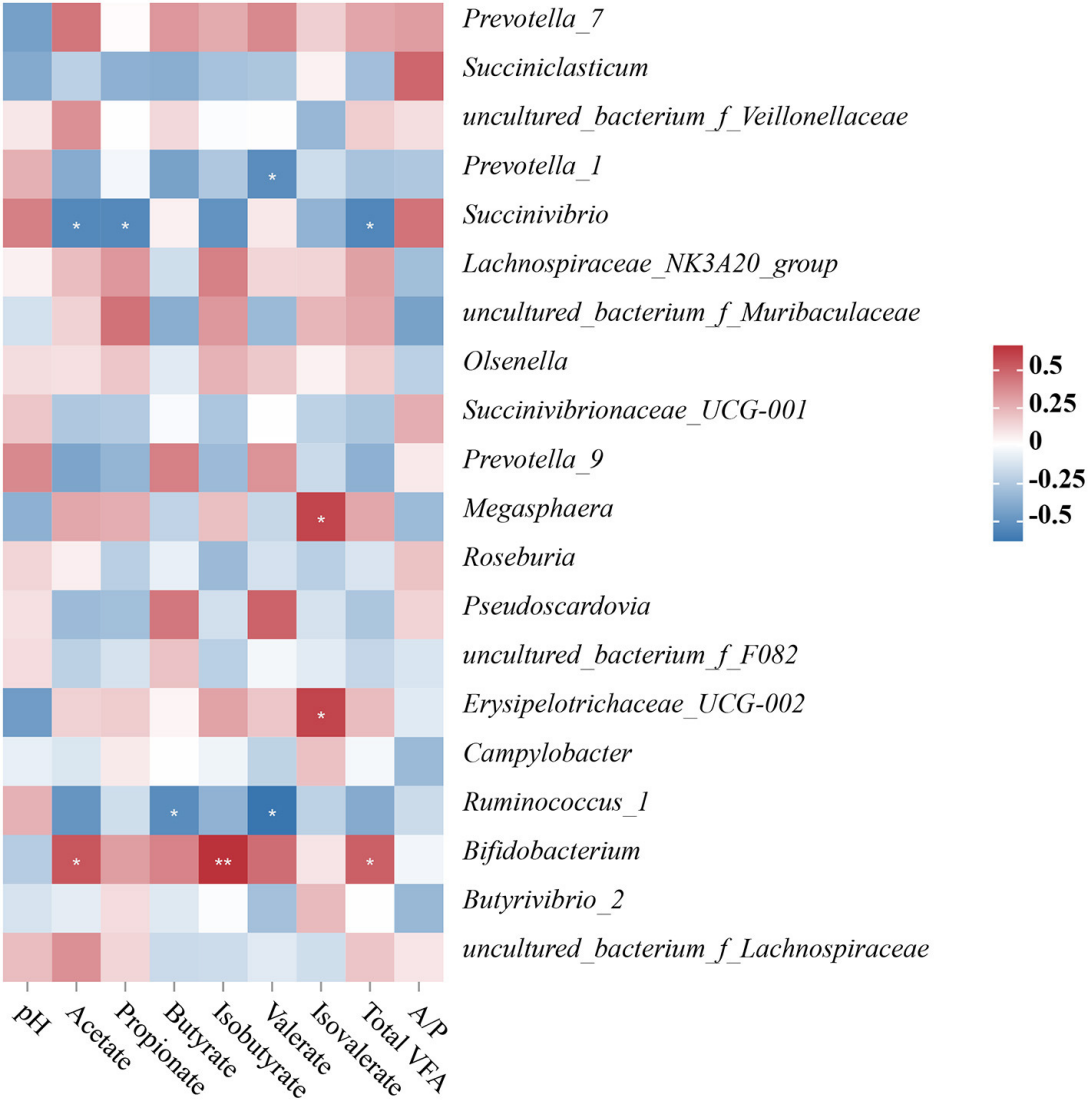
Changes in relative mRNA expression of genes related to the tight junction of ruminal tissue in sheep with normal chow diet (NCD), high-fat diet (HFD) and HFD diet supplemented in spirulina (HFS + S). “^*^” represents a significant correlation (*P* < 0.05), “^**^” represents an extremely significant correlation (*P* < 0.01).

As shown in Figure 6, the mRNA expression of tight junction proteins *Claudin-1, Claudin-4* and *Occludin* in rumen were significantly decreased (*P* < 0.01) by high-fat diets compared with the NCD, while these were significantly increased by spirulina supplementation in the HFD + S group (*P* < 0.05).

## Discussion

The rumen's development and morphology have an impact on lambs' digestibility and growth performance and could be measured by rumen weight and rumen papilla length (27, 28). There are several reports on nutrient levels or the composition of feeds that can affect the rumen histological morphology of ruminants (29–31). In the present study, compared with the control group, the ruminal papilla length, thickness of total epithelial, stratum corneum, and stratum granulosum were significantly lower in the HFD group, while the rumen's histological morphology showed no significant difference in the NCD and HFD + S groups, which indicated that 3% spirulina supplementation could ameliorate rumen disorder caused by an HFD diet. This result is also similar to a previous study in which HFD leads to damage to the digestive tract (32). Rumen pH and VFA are both important pointers of rumen fermentation in ruminants, which can reflect rumen wall function and regulate the internal environment acid–base balance (33, 34). In this study, there were no significant variations in pH across the three groups. This is most likely because the rumen wall has a restricted capacity to affect VFA transfer as well as the ability of ruminant saliva to dilute pH. Previous studies have shown that lipid supplementation did not affect the pH in the rumen, which might indicate that fiber digestion cellulolytic processes were unaffected or that microbes had adapted to the diet (35, 36). VFA played an important role in promoting rumen development (37). Thus, we analyzed the VFA concentrations in ruminal digesta. The results showed that HFD decreased the total VFA, acetate, propionate, butyrate and valerate concentrations in the rumen, while they were increased by spirulina supplementation. Moreover, it has been recognized that VFA, a major product of microbial fermentation, has a wide range of effects on host physiology (38). Previous studies indicated that the type of feed, time of weaning, and microorganisms all have a role in the rumen growth process (39–41). The results of rumen weight, rumen papillae length, and thickness of ruminal epithelia showed that HFD inhibited rumen development in lambs and that spirulina supplementation into the HFD diet could alleviate it, which were consistent with previous studies (39, 40, 42, 43).

This research used 16S rRNA V3–V4 high-throughput sequencing technology to assess the microorganism diversity in high-fat diets supplemented with spirulina, which not only obtained relatively complete bacteria information but also reduced their separation and cloning error (27). In the present study, HFD changed the richness of rumen bacteria, which was confirmed by community richness estimates according to ACE and Chao1, while community diversity markers like Shannon were not affected. Meanwhile, the OTUs results suggested that HFD and spirulina supplementation could reduce the richness and maintain the relatively mature structure of the microbiota in the rumen of lambs. The Venn group figures and OTUs unweighted UniFrac PCoA further indicated differences in bacterial communities among the three groups, showing that HFD changed the composition of the ruminal digesta bacterial community. This change may be caused by a decrease in total VFA, leading to changes in the ruminal digesta bacterial community. Lipid supplementation altered the concentration of all quantified VFA in the rumen, which was triggered by a larger intake of total fatty acid (44).

The effect of hydrogenated fat on rumen microbiota is controversial. Some studies reported that the addition of saturated fatty acids had no significant effect on rumen microbiota (45–47), while some studies believed that excessive lipid supplementation would have adverse effects on rumen (19, 48). This may be due to the toxicity of large amounts of unsaturated fatty acids produced by the decomposition of excess fat in the rumen to microbiota especially cellulolytic bacteria. In this study, excessive addition of fat powder had a negative effect on rumen microbiota. It is worth noting that in this study, in order to ensure the same level of other nutrients (crude protein), more corn was added to the diet of the high-fat group. In our results, the adverse effects of the high-fat group on the rumen microbiota may also be caused by this reason. Previous studies have also reported that high-concentrate diet will damage the structure of the rumen microbiota and affect the rumen function (49, 50).

Studies have shown that in mammals, especially in ruminants, *Firmicutes, Bacteroidetes, Actinobacteria*, and *Proteobacteria* are the dominant bacteria (51, 52). The dominant bacteria analyzed were also similar in our study with other previous reports (53, 54). The level of *Firmicutes* in the NCD group was significantly higher, while the level of *Proteobacteria* was significantly lower than in the HFD and HFD + S groups. *Firmicutes* are the main bacteria that decompose fiber, including many bacteria that can decompose cellulose, and *Proteobacteria* is a sign of intestinal bacteria imbalance (55, 56). This result indicated that HFD might have a negative influence on rumen fiber digestion and ruminal microbiota balance. The relative abundances of *Megasphaera* and *Prevotella_9* rose at the genus level in HFD-fed sheep. Several processes, including protein metabolism, carbohydrate metabolism, and lipid metabolism, were shown to be favorably linked with several *Prevotella* strains (57). Increases in energy content generated by lipid supplementation in the dietary treatments may be one explanation for the rise in *Prevotella* percentage in the HFD and HFD+S groups in this study. *Megasphaera* was well-known for being a powerful lactate utilizer in the rumen and for helping to avoid lactic acidosis (58–61). In addition, LEfSe analysis showed that the increase in the abundance of *Megasphaera* in the HFD and HFD+S groups further explained why feeding on a high-fat diet did not decrease ruminal pH.

This study performed a correlation analysis between the ruminal bacterial community and the fermentation parameters at the genus level. For example, *Succinivibrio* was adversely linked to total VFA, acetate, and propionate levels. *Succinivibrio*, as the producer of succinate and acetate, can be converted to propionate, which promotes the formation of bacterial proteins (27, 62, 63). *Succinivibrio* is a member of the Succinivibrionaceae family, and its main constituent is succinate, which is a precursor to propionate and acetate (64). Moreover, propionate is an important precursor of gluconeogenesis. A small amount of propionate absorbed by rumen epithelium is converted into lactic acid, and the rest enters the liver to generate glucose through gluconeogenesis or enters the tricarboxylic acid cycle for oxidation. A recent study reported that rumen epithelium development may be improved by propionate as a signaling molecule (65). In this study, the content of propionate in the rumen was consistent with the development trend of the rumen. Also, extensive studies have shown that dietary additives can effectively change rumen fermentation mode, promote propionic acid production, and inhibit methanogenesis (66, 67). According to other studies, spirulina is high in gamma-linolenic acid, and adding it to diet may effectively decrease methane production while increasing propionate synthesis (6, 68). In this study, the addition of spirulina significantly reduced acetate and propionate, but the relationship between spirulina and methane production needs to be further explored. *Erysipelotrichaceae_UCG-002* and *Megasphaera* were positively correlated with the concentration of isovalerate. Previous studies found that members of the Erysipelotrichaceae family had a strong positive correlation with host cholesterol metabolites and high-fat or Western diet–feeding mice (18, 69–71). *Bifidobacterium* is a key probiotic for maintaining intestinal microbial equilibrium, forming a healthy gut barrier and lowering lipopolysaccharide levels (72, 73). Consistent with previous reports, *Bifidobacterium* was inversely associated with a high-fat diet (74–77). Furthermore, *Bifidobacterium* was positively connected to isobutyrate, total VFA, and acetate concentrations in the current investigation. These findings were consistent with prior research that indicated a link between *Bifidobacterium* and VFA (78, 79). Numerous studies have shown that VFAs are an important energy source for ruminants (80, 81). As a result, it was critical to investigate VFA metabolism, especially butyrate, which is also recognized as a rumen development stimulator (82, 83). Also, a previous study reported that rumen papillae width and length are the most significant pointers for rating rumen development (84). This study's results showed that HFD feeding reduced the content of butyrate in the rumen and inhibited the growth of the rumen papilla, but spirulina supplementation effectively alleviated this phenotype. Another study showed that butyrate could also promote the increase of rumen weight (85). In this study, the difference in rumen weight was not significant among the three groups. However, compared with the HFD group, rumen weight in the HFD+S group showed an upward trend.

Our findings also demonstrated that the HFD reduced *Claudin-1, Claudin-4*, and *Occludin* mRNA expression. Previous research has shown that VFA can increase the expression of *Claudin-1, Claudin-4*, and *Occludin* in the rumen epithelium, encouraging rumen papilla growth (86). These findings also support the deleterious effects of the HFD on rumen function and the beneficial benefits of spirulina supplementation. As a result of these observations, the HFD group's tight junction impairment might be due to rumen bacteria structural disorder, decreased fermentation function, and lower VFA concentration.

In conclusion, this study showed that HFD decreased the length of rumen papillae and total ruminal VFA concentrations in sheep and that spirulina supplementation could effectively alleviate these negative effects. This might be associated with the change in ruminal microbiota composition in sheep including the significant change in the relative abundance of *Megasphaera* and *Prevotella_9*. These results suggest beneficial effects of 3% spirulina supplementation on altering the diversity of ruminal microbiota. This study is meaningful for further exploring the regulation of rumen development and microbiota by spirulina supplementation in an HFD-fed diet.

## Data availability statement

The original contributions presented in the study are publicly available. This data can be found at: NCBI, PRJNA513129 and SRP354618.

## Ethics statement

The animal study was reviewed and approved by Ethics Committee of Nanjing Agricultural University, China. Written informed consent was obtained from the owners for the participation of their animals in this study.

## Author contributions

ZWa and YL conceived and designed the experiments and wrote the paper. ZWa, YB, XY, and JL performed the experiments. ZWa analyzed the data. YZ and FW helped perform the analysis and with con-strictive discussions. ZWe and DW helped conducting the experiments. All authors read and approved the final manuscript.
