# Supplementary material for: Dietary spirulina supplementation modifies rumen development, fermentation and bacteria composition in Hu sheep when consuming high-fat dietary
Source: Front Vet Sci. 2023 Jan 30;10:1001621. doi: 10.3389/fvets.2023.1001621 (PMC9926970; doi:10.3389/fvets.2023.1001621)
Supplement: Supplementary file 1 [file Data_Sheet_1.docx]

***Table S1***. Primer sequences and amplicon information

| Gene |  | Primers (5’-3’) | Bp | Accession |
| --- | --- | --- | --- | --- |
| Occludin | Forward | GCCTGTGTTGCCTCCACTCTTG | 1569 | XM_015101255.3 |
|  | Reverse | CCGTAGCCATAACCATAGCCATAGC |  |  |
| CLDN-1 | Forward | GTCTTTGGGGGCGTGATCTT | 150 | NM_001185016.1 |
|  | Reverse | CCAGCCAATGAAGAGAGCCT |  |  |
| CLDN-4 | Forward | CACGCAACAACAAGCCCTAC | 288 | NM_001185017.2 |
|  | Reverse | GTGGCCCAGGAGTCTCTTTC |  |  |
| ACTB | Forward | CGCAAGTACTCCGTGTGGAT | 146 | NM_001009784.3 |
|  | Reverse | TAACGCAGCTAACAGTCCGC |  |  |

***Table S2.*** Number of sequences, estimated sample coverage, diversity and OTU richness at 3 % dissimilarity level in each sample.

| Sample ID | Raw Reads | Clean Reads | Effective Reads | AvgLen(bp) | GC(%) | Q20(%) | Q30(%) | Effective(%) |
| --- | --- | --- | --- | --- | --- | --- | --- | --- |
| NCD1 | 80218 | 78523 | 73549 | 422 | 54.39 | 97.75 | 93.58 | 91.69 |
| NCD2 | 79754 | 78226 | 75312 | 417 | 55.3 | 97.68 | 93.45 | 94.43 |
| NCD3 | 79694 | 78345 | 77936 | 418 | 54.17 | 97.88 | 93.94 | 97.79 |
| NCD4 | 80082 | 78517 | 74036 | 422 | 53.91 | 97.84 | 93.78 | 92.45 |
| NCD5 | 80164 | 78833 | 75604 | 418 | 53.69 | 97.76 | 93.58 | 94.31 |
| HFD1 | 79774 | 78539 | 72349 | 422 | 54.27 | 97.9 | 93.83 | 90.69 |
| HFD2 | 79878 | 78605 | 71974 | 419 | 55.24 | 97.88 | 93.88 | 90.1 |
| HFD3 | 79574 | 78235 | 71988 | 420 | 54.56 | 97.83 | 93.78 | 90.47 |
| HFD4 | 80082 | 78905 | 74080 | 418 | 53.92 | 97.97 | 94.06 | 92.51 |
| HFD5 | 80441 | 78940 | 72104 | 418 | 53.78 | 97.83 | 93.79 | 89.64 |
| HFDS1 | 80066 | 78935 | 73036 | 422 | 54.17 | 98.05 | 94.13 | 91.22 |
| HFDS2 | 79581 | 78169 | 69828 | 419 | 55.78 | 97.81 | 93.67 | 87.74 |
| HFDS3 | 79748 | 78381 | 73147 | 422 | 54.13 | 97.91 | 93.9 | 91.72 |
| HFDS4 | 79762 | 78530 | 71853 | 422 | 53.56 | 97.87 | 93.78 | 90.08 |
| HFDS5 | 79945 | 78537 | 74537 | 423 | 54.51 | 97.67 | 93.34 | 93.24 |
